# Plant 3’ Regulatory Regions From mRNA-Encoding Genes and Their Uses to Modulate Expression

**DOI:** 10.3389/fpls.2020.01252

**Published:** 2020-08-14

**Authors:** Willian Souza Bernardes, Marcelo Menossi

**Affiliations:** Functional Genome Laboratory, Department of Genetics, Evolution, Microbiology, and Immunology, Institute of Biology, University of Campinas - UNICAMP, Campinas, Brazil

**Keywords:** molecular biotechnology, plant 3’ regulatory regions, plant *cis*-regulatory elements, increase gene expression in plants, cleavage and polyadenylation factors in plants

## Abstract

Molecular biotechnology has made it possible to explore the potential of plants for different purposes. The 3’ regulatory regions have a great diversity of *cis*-regulatory elements directly involved in polyadenylation, stability, transport and mRNA translation, essential to achieve the desired levels of gene expression. A complex interaction between the cleavage and polyadenylation molecular complex and *cis*-elements determine the polyadenylation site, which may result in the choice of non-canonical sites, resulting in alternative polyadenylation events, involved in the regulation of more than 80% of the genes expressed in plants. In addition, after transcription, a wide array of RNA-binding proteins interacts with *cis*-acting elements located mainly in the 3’ untranslated region, determining the fate of mRNAs in eukaryotic cells. Although a small number of 3’ regulatory regions have been identified and validated so far, many studies have shown that plant 3’ regulatory regions have a higher potential to regulate gene expression in plants compared to widely used 3’ regulatory regions, such as *NOS* and *OCS* from *Agrobacterium tumefaciens* and *35S* from cauliflower mosaic virus. In this review, we discuss the role of 3’ regulatory regions in gene expression, and the superior potential that plant 3’ regulatory regions have compared to *NOS, OCS* and *35S* 3’ regulatory regions.

## Introduction 

In eukaryotes, nuclear processing of pre-messenger 3’ RNA (pre-mRNA 3’) influences several subsequent stages of gene expression, that include, but are not limited to mRNA splicing, stability, transport and translation (Zhao et al., 1999; Millevoi and Vagner, 2010). A highly efficient surveillance system degrades any pre-mRNA that has not been properly processed (Jensen et al., 2001). In mammalian and yeast, a molecular complex composed of more than 20 proteins, interacts with *cis-*elements present in the pre-mRNA 3’ to cleave and polyadenylate the newly transcribed mRNA (Mandel et al., 2008; Chan et al., 2011). These *cis-*elements are polyadenylation signals, which define where the molecular complex should cleave and polyadenylate the pre-mRNA (Loke et al., 2005).

In plants, a similar molecular complex has been identified (Hunt, 2008; Hunt et al., 2012), and although less conserved, plants also have polyadenylation signals: far upstream element (FUE), near upstream element (NUE) and the cleavage element (CE) (Xing and Li, 2011). The polyadenylation site (PAS), point from which the pre-mRNA is cleaved and polyadenylated, is defined by surrounding *cis-*elements (Tian and Manley, 2016). Furthermore, the strength of a given PAS is also defined, in part, by the *cis-*elements (Neve et al., 2017). Polyadenylation is essential for the stability of the transcript, preventing the mRNA from being the target of posttranscriptional gene silencing (PTGS) *via* RNA-dependent RNA polymerase 6 (RDR6) in plants (Luo and Chen, 2007). Besides, polyadenylation mediates transcriptional processes such as initiation, elongation, and termination (Mapendano et al., 2010), as well as post-transcriptional processes, such as transport of mRNA into the cytoplasm and start of translation (Millevoi and Vagner, 2010; Chan et al., 2011).

Eukaryotes have a mechanism termed alternative polyadenylation (APA), which allows the selective use of PAS in genes containing multiple PAS. The APA allows fine regulation of gene expression, being recognized as one of the main regulatory mechanisms of expression (Hunt, 2012; Tian and Manley, 2016). It is estimated that approximately half of the eukaryotic genes have multiple PAS (Tian et al., 2005; Shen et al., 2008a; Hunt, 2012). Through APA, a single gene containing multiple PAS can generate a considerable number of transcript isoforms, thereby producing a highly diversified transcriptome (Tian and Manley, 2013). The availability of multiple PAS in the same 3’ regulatory region enables the inclusion or exclusion of 3’ untranslated region (3’UTR) sequences, resulting in transcripts that may differ in particularities involving post-transcriptional processes such as stability, transport and translation (Mayr, 2016), and even protein localization (Berkovits and Mayr, 2015). Moreover, several studies have shown that APA activity can vary according to the cell state or cycle, as well as with the cell type, being involved in different biological processes in plants (Xing and Li, 2011; Deridder et al., 2012; Tian and Manley, 2016; Ji et al., 2018).

The non-coding regions downstream of coding sequences (CDS) is usually termed terminators. However, we believe 3’ regulatory region is a more appropriate term, as it will be referred to here. This is because transcription termination is only one of the roles of the 3’ regulatory regions, which, in many cases, can have profound effects on gene expression, as it will be discussed in this review (Menossi et al., 2003; Yang et al., 2009; Nagaya et al., 2010; Hirai et al., 2011; Hiwasa-Tanase et al., 2011; Matsui et al., 2014; Diamos and Mason, 2018; Pérez-González and Caro, 2018; Rosenthal et al., 2018; Yamamoto et al., 2018).

## 3’ Regulatory Regions

**Figure 1:**
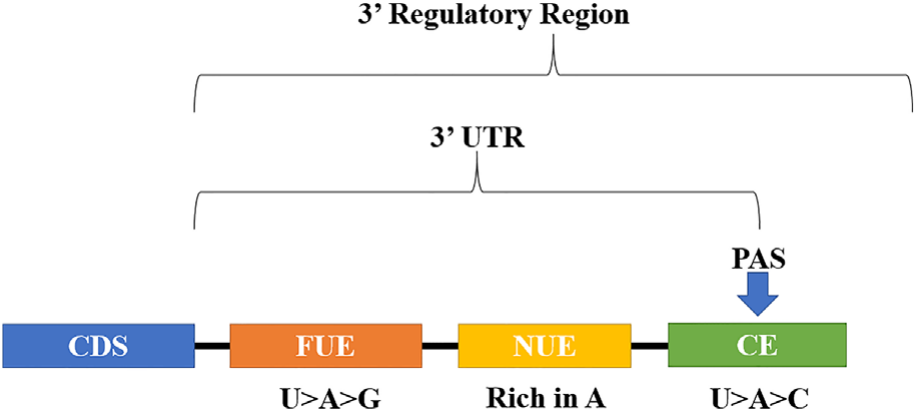
Structure of the tripartite polyadenylation signal in plants: FUE, far upstream element; NUE, near upstream element; CE, cleavage element; PAS, polyadenylation site; CDS, coding sequence; 3’UTR, 3’ untranslated region.

Eukaryotes have non-coding DNA sequences located downstream of the CDS, termed 3’ regulatory regions (**Figure 1**), which are involved in important processes of gene transcription termination, such as cleavage and polyadenylation (Ingelbrecht et al., 1989; Huang and Carmichael, 1996; Luo and Chen, 2007; Rosenthal et al., 2018). Although they do not encode polypeptides, 3’ regulatory regions have *cis*-elements that guide the CPMC during cleavage and polyadenylation (Yang et al., 2009; Xing et al., 2010; Hiwasa-Tanase et al., 2011; Hunt et al., 2012; Matsui et al., 2014; Rosenthal et al., 2018). Indeed, 3’ regulatory regions have a significant weight on gene expression levels, as shown by the use of different 3’ regulatory regions in expression cassettes (Ingelbrecht et al., 1989; Mitsuhara et al., 1996; Richter et al., 2000; Nagaya et al., 2010; Hirai et al., 2011; Diamos et al., 2016; Wei et al., 2017; Diamos and Mason, 2018; Rosenthal et al., 2018). Some 3’ regulatory regions have multiple PAS, being largely responsible for the diversity of the eukaryotic transcriptome (Xing et al., 2010; Xing and Li, 2011; Tian and Manley, 2013). The presence of multiple PAS in the same 3’ regulatory region allows fine regulation of gene expression through the APA mechanism, with consequent effects on mRNA metabolism and metabolic pathways (Wei et al., 2017; Hong et al., 2018; Turner et al., 2018; Wang et al., 2019). However, the mechanism that controls the use of a particular PAS over another is a complex process that is far from being wholly understood (Neve et al., 2017; Turner et al., 2018).

## *Cis-*Elements in Plants

Plants, as well as mammals, yeast and algae have regulatory sequences, termed *cis*-elements that guide pre-mRNA polyadenylation (Loke et al., 2005; Hunt, 2008; Mandel et al., 2008; Shen et al., 2008b). In plants, a tripartite model has been suggested, composed by the *cis-*elements, far upstream element (FUE), near upstream element (NUE), and cleavage element CE, which together constitute the signal of polyadenylation (**Figure 1**). Rich in U>A>G, the FUE element has 6-18 nucleotides (nt) in size, distributed within a region of approximately 125 nt, which in general starts at approximately 30 nt upstream of PAS. The NUE element is A-rich, with a size ranging from 6–10 nt, and is located approximately between 10 to 40 nt upstream of the PAS. The CE element is composed of a region rich in U>A>C, having as part of its sequence the cleavage site itself, where after the RNA cleavage the polyadenine tail is added, in a process called polyadenylation. The cleavage site is formed by 2 nt, defined by some authors as YA, where Y = U or C (Li and Hunt, 1997; Loke et al., 2005; Hunt, 2008; Xing and Li, 2011).

Plants have NUE sequences similar to the dominant A(A/U)UAAA polyadenylation signal found in mammals. Although found in more than 50% of mammalian genes, AAUAAA, the most common variant of hexamer is found in only 10% of transcripts from *Arabidopsis thaliana* and rice (*Oryza sativa*) (Zarudnaya et al., 2003; Chan et al., 2011; Xing and Li, 2011). Also, a recent analysis of bioinformatics found that AAUAAA is also very poorly conserved in mosses (*Selaginella moellendorffii* and *Physcomitrella patens*), not exceeding 8%, with the first 2 nt being highly degenerate (Zhao et al., 2019). Also, the second most frequent variant AUUAAA hexamer in mammals, present in about 16% of transcripts, was found in only 2.17% and 2.08% of *A. thaliana* and *S. moellendorffii*, respectively (Zhao et al., 2019). Unlike mammals, point mutations in the AAUAAA variant have little influence on the efficiency of polyadenylation in plants, with some mutations even increasing, which justifies the low frequency of this exact sequence in plants (Rothnie et al., 1994). However, *in vitro* assays showed that the deletion of NUE or FUE results in the choice of unusual PAS (Zhao et al., 2011), consistent with findings from other earlier studies (Mogen et al., 1990; Rothnie et al., 1994).

The sequence complementarity profile that *cis*-elements present can generate secondary structures in the 3’ end region of the pre-mRNAs that appear to positively influence the functionality of the *cis-*elements itself (Loke et al., 2005). The formation of these secondary structures are important for the interaction between proteins from polyadenylation complex and mRNA (Zarudnaya et al., 2003). The efficiency of recognition and choice of a given PAS is partially determined by the signal strength of the *cis*-elements (Loke et al., 2005; Guo et al., 2016; Neve et al., 2017). More importantly, these *cis*-elements can be found in different regions of the genes, such as 5’UTR, exons, introns, and mainly in 3’ regulatory regions (Simpson et al., 2003; Hunt, 2012; Hoque et al., 2013; Rosenthal et al., 2018).

## Cleavage and Polyadenylation Molecular Complex

During the final steps of the transcription process in eukaryotes, a multiprotein complex composed of more than 20 proteins, here named cleavage and polyadenylation molecular complex (CPMC), indispensable for the biogenesis of mRNA, recognizes and interacts with the *cis*-elements to cleave and polyadenylate the pre-mRNA (Chan et al., 2011). Most of these proteins have already been identified in animals and yeasts (Mandel et al., 2008), and most of their homologs have also been identified in plants (Hunt, 2008; Zhao et al., 2009; Zhao et al., 2011). This molecular complex is formed by subcomplexes, also called factors, and can be divided basically into four large subcomplexes: Cleavage and Polyadenylation Specificity Factor (CPSF), Cleavage Stimulatory Factor (CstF), Cleavage Factor I e II (CF I e II) (Millevoi and Vagner, 2010; Chan et al., 2011). Each factor is formed by protein subunits that interact with each other in the form of even more complex heterodimers, heterotrimers or oligomers. The interaction between the protein subunits and the *cis*-elements is crucial to maintain the cohesion of the complex (Chan et al., 2011). The assembly of the molecular complex takes place after binding of these factors to the *cis*-elements, which generally takes around 10 seconds, with this response time being influenced by the strength of the PAS (Chao et al., 1999).

The best and richest description of the molecular polyadenylation complex among eukaryotes is by far that of mammals. In mammals, the CPSF factor mediates the cleavage and polyadenylation process. After recognizing the canonical hexamer A(A/U)UAAA, or its more frequent variant, AAUAAA, CPSF recruits the other factors to cleave and polyadenylate the 3’ pre-mRNA region. There is evidence indicating that CPSF160 is the subunit responsible for recognizing A(A/U)UAAA (Murthy and Manley, 1995) and recently it has been shown that CPSF30 and WDR33 interact directly with the AAUAAA hexamer (Chan et al., 2014). A cryogenic electron microscopy study reported that in humans, CPSF160 does not interact with the AAUAAA, but functions as a scaffold to preorganize two other subunits, CPSF30 and WDR33, which synergistically bind to the hexamer with high affinity (Sun et al., 2018).

Indeed, AtCPSF30 has also been shown to be an RNA-binding protein (RBP) with an affinity for U-rich sequences such as FUE (Hunt, 2008). Assays with orthologs of *AtCPSF30*, encoding CPSF160 and WDR33 in *A. thaliana* (*AtCPSF160* and AtFY, respectively), found an interaction between these subunits, demonstrating that a similar complex may form in plants during the processing of the 3’ end pre-mRNA (Zhao et al., 2009). AtCPSF30 is involved in the choice of canonical NUE from a large number of genes in *A. thaliana*, and mutations of this subunit result in the choice of unusual PAS (Thomas et al., 2012). AtFY was found to be involved in the 3’ end processing of mRNA in *A. thaliana* (Simpson et al., 2003) and recently, the role of AtFY in the recognition of canonical NUE has been demonstrated using *fy* mutants in *A. thaliana* (Yu et al., 2019). Indeed, the choice of canonical NUE appears to rely on the interaction between AtCPSF30 and AtFY, with double mutants being able to generate up to 50% more APA events, which had some interference on processes such as the control of flowering time (Yu et al., 2019). These data are in agreement with previous studies (Jiang et al., 2012; Chakrabarti and Hunt, 2015). Besides, AtCPSF160 and AtCPSF30 have been shown to possess nuclear localization (Delaney et al., 2006; Xu et al., 2006), the nuclear location of AtCPSF30 appears to depend on its interaction with AtCPSF160 (Rao et al., 2009).

In *A. thaliana*, *At1g30460* is the gene that encodes AtCPSF30, which interestingly is the target of alternative splicing events, resulting in the production of another protein containing an additional motif related to pre-mRNA splicing in mammals. These two proteins may form different complexes, connecting mRNA splicing and polyadenylation in plants (Delaney et al., 2006). AtCPSF30 has been shown to possess endonuclease activity, which appears to be inhibited by AtFip1, a mammalian hFip1 orthologous protein (Addepalli and Hunt, 2007). Furthermore, *AtCPSF30* mutants of *A. thaliana* are more tolerant to oxidative stress, confirming a likely role in the regulation of gene expression (Zhang et al., 2008).

As previously demonstrated, CPSF73 has endoribonuclease activity and may be the subunit responsible for pre-mRNA cleavage in humans (Mandel et al., 2006). Two mammalian CPSF73-like proteins, AtCPSF73(I) e AtCPSF73(II), are found in *A. thaliana* (Xu et al., 2004). Although very similar, AtCPSF73(I), and AtCPSF73(II) have distinct roles in plants (Xu et al., 2004; Xu et al., 2006). An *in vitro* cleavage assay showed that the AtCPSF73(I) subunit has endonuclease activity (Zhao et al., 2011).

In mammals, *in vitro* experiments suggest that after cleavage of the pre-mRNA, CPSF160 and hFip1 act directly in the recruitment of poly-A polymerase (PAP), protein responsible for polyadenylation (Barabino et al., 1997; Kaufmann et al., 2004). The expression of hFip1 changes according to the degree of cell differentiation, and it has been shown to be a potent regulator of APA. hFip1 promotes stem cell maintenance by activating APA profiles specific to embryonic stem cells (ESC), and also restores APA profiles similar to those found in ESC during the reprogramming of somatic cells (Lackford et al., 2014). Intriguingly, it has recently been proposed that CPSF can mediate cytoplasmic polyadenylation of mRNAs (Dai et al., 2019).

In *A. thaliana*, there is an interaction between the counterparts of hFip1 and PAP, shown by a yeast two-hybrid assay between AtFip1 with AtPAP. Also, AtFip1 stimulates AtPAP activity, and as well as hFip1, AtFip1 is also an RBP with a preference for G-rich sequences (Forbes et al., 2006). PABPN1 (poly-A polymerase-binding nuclear protein) is another critical piece of PAP activity. This protein stimulates the reaction of PAP catalysis during the synthesis of the polyadenine tail and also dictates its size by regulating the interaction between CPSF and PAP (Kühn et al., 2009). In *A. thaliana*, at least three isoforms of PABPN, AtPABN1, 2, and 3 are found, and interaction between AtPABN and AtPAP isoforms is reported (Hunt et al., 2008). Also, the interaction between AtPABN1, AtPAP4, and AtCPSF30 has been reported (Forbes et al., 2006).

In mammals, the CstF factor contributes decisively to the processing of the 3’ regulatory region of the pre-mRNA. However, the stable binding of the CstF subcomplex to the 3’ regulatory region of the pre-mRNA is dependent on the interaction with the CPSF subcomplex. An interaction between CstF64 and CPSF160 proved to be necessary to define the cleavage site (Chan et al., 2011). CstF64 is directly involved in the recognition of PAS and in the global regulation of APA, being able to binding to G/U-rich sequences downstream of the PAS, and its specificity dependent of the interaction with CPSF (Yao et al., 2012; Masoumzadeh et al., 2020). Also, CstF77 increases the affinity of CstF64 for RNA targets, recruiting CstF50, that is involved in the recognition of G/U-rich sequences (Yang et al., 2018). Once in the cytoplasm, the transport of CstF64 to the nucleus is dependent on its interaction with CstF77, essential for the both cleavage and polyadenylation (Grozdanov et al., 2018b).

It was demonstrated *in vitro* that CstF64 is essential for an adequate differentiation of embryonic stem cells into endodermal lines, and its absence may result in dysfunctional cardiomyocytes (Youngblood and Macdonald, 2014). Furthermore, co-expression of CstF50 or CstF77 with CstF64 promoted an increase in cleavage and polyadenylation rates of a reporter gene *in vitro* (Grozdanov et al., 2018b). CstF64/tau is encoded by *Cstf2t*, a paralog of the *Cstf2* gene that encodes CstF64. *Cstf2t* is expressed in germ cells, also has a role on polyadenylation, being indispensable for spermatogenesis in rats (Harris et al., 2016; Grozdanov et al., 2018a). CstF64/tau binds to sequences rich in U>G also downstream of the PAS (Mandel et al., 2008). In addition, CstF64/tau promotes the use of non-canonical distal PAS, an important regulator of the APA (Hwang H. et al., 2016).

*A. thaliana* has orthologs of subunits CstF64 and CstF77, encoding AtCstF64 and AtCstF77, that interact *in vitro* and have the ability to bind RNA, as shown for mammalian counterparts (Yao et al., 2002; Bell and Hunt, 2010). In *Glycine max* there is gene duplication for *CstF50* and *CstF64*, probably due to recent genomic duplication. In contrast, *S. moellendorffii* presents two orthologs for *CstF64*. The presence of orthologs for *CstF50* in some plants and the absence in others, such as *Chlamydomonas reinhardii* and *Populus trichocarpa*, suggests that there may be functional redundancy (Hunt et al., 2012). Interestingly, *A. thaliana* also has a protein, enhanced silencing phenotype 1 (ESP1), that has a degree of similarity with mammalian CstF64. Unlike AtCstF64, ESP1 does not have the canonical RNA (RRM) recognition domain present in CstF64. However, similarly to AtCstF64, ESP1 presents the domain that allows an interaction with CstF77 and other factors involved in cleavage and polyadenylation (Herr et al., 2006).

The subcomplex CFIm is indispensable for the cleavage step of pre-mRNA in mammals (Ryan, 2007; Chan et al., 2011). CFIm also seems to be involved in the choice of PAS, according to APA events presented by HeLa cells knocked down for CFIm25 (Kubo et al., 2006), what seems to be happening also for CFIm68 (Kim et al., 2010). In mammals, CFIm presents itself as a heterotetrametric complex composed of two CFIm25, one CFIm59 and one CFIm68 subunits. However, it was demonstrated *in vitro* that the complex presents activity only with a CFIm25 dimer and two CFIm68 subunits, suggestive of functional redundancy between CFIm59 and CFIm68 (Ruëgsegger et al., 1998), although mutants for CFIm68 have effects on APA while CFIm59 does not (Kim et al., 2010). CFIm assists in the interaction of PAP with hFip1 and CPSF160 during the cleavage process. CFIm also assists in the definition of PAS and potentiates the recognition of noncanonical *cis*-elements (Chan et al., 2011), being involved in the recruitment of hFip1 and PAP (Venkataraman et al., 2005). CFIm binds specifically to the *cis*-elements UGUA, with CFIm25 being the subunit responsible for the recognition and interaction with UGUA (Yang et al., 2010).

As demonstrated by mutation studies, the RRM of CFIm68 increases the affinity of CFIm25 for UGUA (Yang et al., 2011a; Yang et al., 2011b). It was demonstrated that CFIm59 and CFIm68 also potentiate the use of PAS that have the polyadenylation signal UGUA, being the position of UGUA able to affect this activity *in vitro*. This CFIm activity requires an interaction with hFip1, mediated by the serine-arginine (RS) repeat domain present in CFIm59 and CFIm68. In addition, the binding of CFIm to UGUA promotes the recruitment of CPSF and CstF (Zhu et al., 2018). In *A. thaliana*, an interaction between AtFip1 with AtPAP, AtCFI25, AtCPSF30 and AtPABN1 was reported, suggesting that AtFip1 and AtPAP may also be recruited by AtCFI during the processing of the 3’ end pre-mRNA region (Forbes et al., 2006). *A. thaliana* has at least two genes encoding CFIm25 orthologs, *At4g25550* and *At4g29820*, and at least four *CFIm68* orthologs have been found in plants (Hunt et al., 2012).

In contrast, little is known about the subcomplex CFIIm, which has two subunits, Clp1 and Pcf11, required for the cleavage process (Chan et al., 2011). Recently it was demonstrated that Pcf11 has a role in the global regulation of APA events, since it promotes the use of proximal PAS, and its depletion increases the use of distal PAS, which seems to be true also for Clp1 (Li et al., 2015; Ogorodnikov et al., 2018). In *A. thaliana*, at least two orthologs were identified for *Pcf11*: *At4g04885* and *At2g36480*. In particular, At4g04885 presents two of the three functional domains found in Pcf11 (Hunt et al., 2012). The depletion of AtPCFS4, a homologue of the yeast Pcf11p, resulted in a delay in flowering time in *A. thaliana*. It was shown that AtPCFS4 is an APA regulator, promoting the use of more proximal PAS, within the intron 3 from the *FCA* gene (Xing et al., 2008b).

Orthologs for the gene encoding the Clp1 subunit also appear to be present in plants, being in *A. thaliana* encoded by two genes, *At3g04680* and *At5g39930* (Hunt et al., 2012). An interaction between AtPCFS4 and AtCLPS3 (*At3g04680*) has already been demonstrated (Xing et al., 2008b). The suspicion that AtCLPS3 is also an APA regulator was raised by the fact that the overexpression of *AtCLPS3* promotes the use of a regular PAS in FCA, which results in the functional FCA isoform, causing early flowering in *A. thaliana* (Xing et al., 2008a). Also, direct interactions between AtCLPS3 and AtFY, AtCPSF30, AtCPSF100 e AtCPSF160, as well as between AtCLPS5 (At5g39930), AtFip3 and AtFip5 were observed (Hunt et al., 2008; Xing et al., 2008a; Xing et al., 2008b).

Although plants exhibit homologous proteins and patterns of interaction between subunits similar to their counterparts in mammals and yeasts, their functions may be different. Another point to consider is that unlike the CPMC of mammals and yeasts, where the subunits are encoded by a single gene, some subunits of *A. thaliana* are encoded by gene families, and, the same gene may encode at least two isoforms of the same subunit. Also, the possibility of gene duplication, mainly highly conserved subunits, may incur functional redundancy or even functional specialization (Hunt, 2008; Zhao et al., 2009; Hunt et al., 2012).

## Polyadenylation

Polyadenylation is the process of synthesis of a polymer of adenine, having been observed for the first time in the 60’s (Edmonds and Abrams, 1960). The polyadenylation process is oriented by *cis*-elements, and involves all CPMC proteins and PAP (Bardwell et al., 1990; Misra and Green, 2016). After PAS recognition and pre-mRNA cleavage by CPMC, the PAP protein initiates the synthesis of the adenine polymer at the 3’ end of the pre-mRNA, from the cleavage site, resulting in a tail of polyadenines (tail poly-A) ranging from 70 to 250 nt between eukaryotes (Kühn et al., 2009). Most mature eukaryotic mRNAs are polyadenylated. It is estimated that less than 5% is not, most of which are histone mRNAs (Tian et al., 2005; Djebali et al., 2012).

*A. thaliana* has at least three canonical nuclear PAPs, AtPAPS1, AtPAPS2 and AtPAPS4, and one cytoplasmic, AtPAPS3 (Addepalli et al., 2004; Hunt et al., 2008; Meeks et al., 2009). Several studies have investigated whether there is functional redundancy between these AtPAPS isoforms (Hunt et al., 2008; Vi et al., 2013; Trost et al., 2014; Kappel et al., 2015; Czesnick and Lenhard, 2016). Encoded by the *At3g06560* gene, AtPAPS3 is a truncated protein, and is involved in the development of pollen (Hunt et al., 2008). AtPAPS1 (*At1g17980*) is responsible for the polyadenylation of a restricted group of pre-mRNAs, involved in the development of the male gametophyte, leaves and flowers, as well as in response to pathogens, ribosome biogenesis and redox homeostasis. In addition, depletion of AtPAPS1 results in shortening of the poly-A tail of a specific group of transcripts from the *SMALL AUXIN UP RNA* (*SAUR*) family, with an evident reduction in the abundance of these transcripts (Trost et al., 2014; Kappel et al., 2015). In cases of *AtPAPS2* (*At2g25850*) and *AtPAPS4* (*At4g32850*), single or double mutants show normal development, suggesting that there may be functional redundancy between these isoforms and *AtPAPS1* (Vi et al., 2013). Interestingly, a more recent study has shown that while *AtPAPS2* and *AtPAPS4* promote flowering, and *AtPAPS1* causes delays in the transition to flowering (Czesnick and Lenhard, 2016).

The polyadenylation process seems to be necessary for the nuclear export of mRNAs (Huang and Carmichael, 1996). Polyadenylation also seems to be extremely important for translation, since the high affinity of poly-A-binding protein (PABP) for A-rich sequences, promotes its association with the poly-A tail, allowing the association of PABP with EIF4G, which then associates with EIF4E, in a sequence of interactions that are crucial for the recruitment of the 40S ribosomal subunit (Jacobson and Favreau, 1983; Tarun and Sachs, 1996; Wells et al., 1998; Cho et al., 2019). More importantly, long poly-A tail mRNAs have higher translation rates than short poly-A tail mRNAs (Beilharz and Preiss, 2007). In addition to mediating transcription termination, the polyadenylation process also likely mediates processes such as initiation, promoting the recycling of proteins involved in transcription (Mapendano et al., 2010), and elongation, as demonstrated by the stable interaction of CPSF with the transcription factor TFIID and with the Pol II elongation complex (Dantonel et al., 1997).

Poly-A tail is also essential for mRNA stability since non-polyadenylation mRNAs are targets of PTGS *via* RNA-dependent RNA polymerase 6 (RDR6) in plants. It has been reported that non-polyadenylated mRNAs are used as templates by RDR6 to produce long double-stranded RNAs (dsRNAs) in plants. These molecules are subsequently used as a substrate by dicer-like enzymes (DCL2 and DCL4), resulting in the production of siRNA by argonaute enzymes and consequently in gene silencing mediated by the RNA-induced silencing complex (RISC) (Dalmay et al., 2000; Baeg et al., 2017). *In vitro* assays have demonstrated that the poly-A tail inhibits the initiation step, not the RDR6 elongation step and that the poly-A tail size is important for this inhibition (Baeg et al., 2017). Indeed, polyadenylated mRNAs have different proteins attached to their 3’ end and poly-A tail. For example, AtPABN acts as an obstacle to RDR6 binding, while other proteins are involved in nuclear export, thereby evading RDR6 action (Luo and Chen, 2007). In fact, non-polyadenylated mRNAs accumulate in the nucleus (Huang and Carmichael, 1996).

Once in the cytoplasm, mRNA is targeted by different exoribonucleases that shorten the poly-A tail, a process called deadenylation, taken as a starting point that leads to two different pathways of mRNA degradation: 5’ deadenylation-dependent decapping and 3’ decay (Meyer et al., 2004). The main pathway of mRNA decay in yeast is the deadenylation-dependent decapping pathway, which involves a molecular complex composed of different proteins, including two deadenylases, CCR4p/POP2p (Tucker et al., 2001). Interestingly, CCR4-POP2 complex can be recruited by Pumilio homologs (PUMs), resulting in increased deadenylation (Weidmann et al., 2014). After deadenylation, mRNA can be targeted by decapping enzymes, such as Dcp1 and Dcp2, responsible for cleavage of the 5’cap, which makes degradation of the 5’ to 3’ mRNA possible by the hydrolytic activity of the Xrn1p exoribonuclease. Once deadenylated, mRNA may also be degraded in the 3’ to 5’ direction by the exoribonucleolytic activity of a multiprotein complex called the exosome, which does not require cleavage of the 5’cap (Coller and Parker, 2004; Meyer et al., 2004). In addition, mRNAs that have lost the poly-A tail are direct targets of repression by PUMs (Etten et al., 2012). As expected, most of the protein involved in the two different pathways of degradation following mRNA deadenylation are also found in plants. Many of these proteins are encoded by gene families, suggesting specialization or functional redundancy (Chiba and Green, 2009).

The size of the poly-A tail may influence the lifetime of the mRNA due to the continuous shortening that the poly-A tail undergoes in the cytoplasm by the action of deadenylases (Eckmann et al., 2011; Weill et al., 2012). However, a recent study presented data that are in contrast to other studies regarding the correlation between poly-A tail size and stability. Analyzing the data set on somatic cell poly-A tail length, it was found that transcripts with short poly-A tail showed higher levels of stability and translation, while transcripts with long poly-A tail showed lower levels of stability and translation (Lima et al., 2017). Changes in the size of the poly-A tail of certain genes during the cell cycle, such as the shortening of the poly-A tail, preventing translation during the M phase (Park et al., 2016). Nevertheless, no correlation was found between the size of the poly-A tail with translation, not even with accumulation of mRNA at steady-state, but, in contrast, transcripts with longer poly-A tails presented greater stability (Chang et al., 2014). As we will see later, other mechanisms than just the length of the poly-A tail are involved with mRNA metabolism.

## Alternative Polyadenylation

Alternative polyadenylation (APA) is a mechanism whereby it is possible to generate transcript isoforms with different 3’UTR or CDS from the same gene, directly reflecting in the diversity of the transcriptome and proteome, and therefore, in the fate of these biomolecules in eukaryotes (Tian and Manley, 2013; Tian and Manley, 2016). APA occurs due to the presence of multiple PAS in the same gene and has been recognized by many authors as one of the main mechanisms of gene regulation (Lutz and Moreira, 2010; Hunt, 2012; Tian and Manley, 2016). Although we have only just begun to unravel the mechanism, we know that APA has temporal (cell and developmental cycle) and tissue specificity, in addition to being involved in different biological processes, such as embryogenesis, gametogenesis, morphogenesis, control of flowering time in plants and control of oncogenes expression in animals (Xing and Li, 2011; Deridder et al., 2012; Tian and Manley, 2016; Ji et al., 2018). Moreover, APA events are also involved with growth and development (Hong et al., 2018), circadian rhythm (Yang et al., 2020), cell signaling (Chakrabarti and Hunt, 2015; Li et al., 2017; Conesa et al., 2020), immunity (Lyons et al., 2013; Ye et al., 2019) and stress response in plants (Zheng et al., 2018; Conesa et al., 2020).

In fact, APA is a potent regulatory agent for gene expression, can affect more than 80% of the genes expresses in a plant (Hunt, 2012). About half of mammalian genes have multiple PAS (Tian et al., 2005), which has also been shown to be true for plants (Shen et al., 2008a; Wu et al., 2011; Hunt, 2012; Wu et al., 2014). In mouse, most PAS are found in the 3’UTR region, but a considerable portion can also be found within intronic regions (Hoque et al., 2013). A similar scenario occurs in plants, and, interestingly, PAS are found even in 5’UTR and coding regions (Hunt, 2012). New bioinformatics approaches based on RNA-seq and other sources, have enabled significant advances in the identification of PAS, which will facilitate the study of the regulation of APA-mediated expression in different plant species (Guo et al., 2016; Wu et al., 2016; Chen et al., 2017; Ye et al., 2018). There is currently a database with PAS sets of at least five different plant species, including *A. thaliana*, *O. sativa*, *Medicago truncatula*, *Trifolium pratense* and *Phyllostachys edulis* (Zhu et al., 2020).

The choice of a PAS is determined by the signal strength given by the *cis*-elements, but not only (Cheng et al., 2006; Shi, 2012; Tian and Manley, 2016; Rosenthal et al., 2018). Mammalian genes that present strong PAS have less frequent or even absent APA events. Furthermore, there is evidence of a relationship with gene function, since genes involved in cell metabolism, morphology, and proliferation are more often targets of the APA mechanism (Wang et al., 2018). However, the distance between competing PAS (Li et al., 2015), the availability and affinity of CPMC for *cis*-elements, as well as protein interference from other pathways can contribute to choosing one given PAS over another (Shen et al., 2008a; Hornyik et al., 2010; Neve et al., 2017).

In mice, high CstF64 expression results in the choice of weaker PAS from IgM mRNA, resulting in the expression of IgM secreted isoform, suggesting involvement in mouse B cell maturation (Takagaki et al., 1996). Still, in mouse cells, hPcf11 and hFip1 promote the use of proximal PAS. On the other hand, CFIm25 and CFIm68, as well as PABPN1 and poly-A-binding protein C1 (PABPC1) promote the use of distal PAS (Li et al., 2015). In humans, a negative feedback mechanism comes into play in the presence of high levels of CstF77, which results in the selection of PAS in an intronic region near the promoter, leading to the generation of truncated transcripts and consequently downregulation of CstF77 (Luo et al., 2013).

In *A. thaliana*, the interaction between AtFY and FCA, a RBP involved in flowering promotion, results in the selection of a PAS within intron 3, leading to the production of a truncated and dysfunctional transcript (Simpson et al., 2003). The selection of PAS in intron 3 is promoted by FCA itself in a manner dependent on high cellular levels, acting as a negative feedback, since the removal of intron 3 results in high levels of FCA and early flowering (Quesada et al., 2003). In addition, AtPCFS4 seems to mediate the choice of a PAS in intron 3 in *A. thaliana* (Xing et al., 2008b). More interesting, the differential use of PAS from the same mRNA encoding AtCPSF30 can generate two distinct proteins. For example, the use of a PAS within an intron results in the production of AtCPSF30, and the use of a more distal PAS results in the production of a mammalian splicing factor-like protein (Delaney et al., 2006). Similarly, the use of two different weak PAS within an intron results in short monofunctional lysine ketoglutarate reductase transcripts in *Gossypium hirsutum*, and is likely to occur also in *A. thaliana*, Z*ea mays* and *Lycopersicon esculentum* (Tang et al., 2002).

The modulation of 3’UTR by APA allows changes in the translational regulation, localization, and stability of mRNAs (Meyers et al., 2004; Tushev et al., 2018). Shortening 3’UTR can make the translation of specific mRNAs more efficient by promoting polysome formation (Chang et al., 2015). Differences in the size of 3’UTR can change the location of mRNAs in the cell (Tushev et al., 2018). Interestingly, the size of 3’UTRs can also directly influence the location of proteins. Short 3’UTRs from *CD47* have been shown to promote the localization in the endoplasmic reticulum and long 3’UTRs promote a membrane localization in a HuR-SET-RAC1-dependent manner. This change in location incurred in different functions for the two CD47 isoforms (Berkovits and Mayr, 2015). In mouse, the production of 3’UTR short through the APA allows greater stability of transcripts, since the shortening results in a smaller number of microRNA target sites (Sandeberg et al., 2008). In *A. thaliana*, the shortening of 3’UTRs of mRNA encoding Rubisco Activase (RCA) in response to heat stress promoted greater transcript stability (Deridder et al., 2012), results that were also found for cotton RCA (Deridder and Salvucci, 2007). On the other hand, two studies found that long 3’UTRs were less targeted by microRNAs (Kim et al., 2014; Agarwal et al., 2015). Similarly, Tushev et al. (2018) found that longer 3’UTRs promote higher stability of mRNAs than short 3’UTRs. Whereas, no difference in stability was found between short and long 3’UTR isoforms of most mouse fibroblast mRNAs (Spies et al., 2013). Intriguingly, long 3’UTRs seem to prevent degradation of mRNAs that have uncommon codons (Mishima and Tomari, 2016).

Although the results are controversial, in addition to the presence or absence of microRNA sites, we must consider that APA generates different 3’UTRs with different *cis*-acting elements that can vary in their composition and diversity according to the size of the 3’UTR, sometimes with stabilizing elements, sometimes with elements destabilizing or both. Moreover, long 3’UTRs can form secondary structures (stem-loop) with stabilizing effects, which perhaps short 3’UTRs do not form. Furthermore, APA events and the diversity of regulatory factors may vary according to cell type or state, tissue type or even with environmental stimulus, as well as the availability and performance of *trans*-regulatory factors (Deridder et al., 2012; Ulitsky et al., 2012; Lackford et al., 2014; Díaz-muñoz et al., 2015; Kim et al., 2016).

## *Cis*-Acting Elements in 3’UTRs

During the final stages of the eukaryotic transcription, the CPMC promotes cleavage and polyadenylation of the pre-mRNA in its 3’ end. These two processes are guided by *cis*-elements, and occurs mainly in 3’ regulatory regions. After cleavage, the part of the 3’ regulatory region that remained in the mRNA is now called 3’UTR (**Figure 1**), comprising from the cleavage site to the stop codon of the CDS. Compared to less complex organisms, 3’UTRs of higher organisms have expanded, reaching an average size that can be almost ten times larger (Jan et al., 2011). 3’UTRs are involved in important post-transcriptional processes, including, but not limited to stability, transport and mRNA translation (Mayr, 2016; Mayr, 2017), in addition to mediating protein localization, and therefore, its function (Berkovits and Mayr, 2015). The effects of 3’UTRs on these processes are mediated by RBPs that bind to a wide variety of specific sequences or *cis*-acting elements present in these regions (Cho et al., 2019). Interestingly, some RBPs may be their expression regulated by the 3’UTRs themselves, perhaps involving the presence of *cis*-acting elements (Tian et al., 2019). It is estimated that the human genome can encode at least 1,500 RBPs (Baltz et al., 2012; Gerstberger et al., 2014). In *A. thaliana*, studies point to somewhere between 1,145 and 1,408 RBPs (Marondedze et al., 2016; Marondedze et al., 2019). Once bound to the mRNA, these RBPs recruit effector proteins that will then determine the fate of the transcript (Mayr, 2016).

The AU-rich element (ARE), has between 50 and 150 nt in size and can present multiple copies of the AUUUA motif, normally found in the 3’UTR (Gutiérrez et al., 1999). In mammals, the ARE element is involved in both the 3’ to 5’ and 5’ to 3’ mRNA decay. Some ARE-binding RBPs can promote recruitment complexes involved in translational decay or silencing, others can inhibit degradation by preventing RBPs involved in decay from interacting with ARE (Garneau et al., 2007; Allen et al., 2013). Site-directed mutagenesis of ARE present in 3’UTRs from her1 was enough to prolong the half-life of reporter mRNAs (Tietz et al., 2020). On the other hand, deletion of a sequence in the 3’UTR of *BCL2* with a high content of AREs promoted instability, resulting in low BCL2 levels in murine B cells (Díaz-muñoz et al., 2015). Interestingly, ARE has the potential to induce exosomal degradation of mRNAs without the need for mediation of other proteins, since RNase domains present in exosomal complex proteins have high affinity for AU-rich sequences (Anderson et al., 2006).

Unlike yeast, mammalian transcripts that present the ARE element in their 3’UTR are rapidly degraded, which is also true for plants. The reason for this is due to a poly-A ribonuclease (PARN), a protein with deadenylase activity present only in multicellular eukaryotes (Gutiérrez et al., 1999; Chiba and Green, 2009). PARN promotes rapid deadenylation of mRNA containing multiple copies of the AUUUA pentamer (Lai et al., 2003). It has been reported in HeLa cells that an interaction between CUG-binding protein 1 (CUGBP1) and PARN, promoted the deadenylation of mRNAs that presented ARE in their sequences (Moraes et al., 2006). Consistent with these findings, mutants for AtPARN showed hyperadenylation of embryonic development-specific mRNA, resulting in slow development, that was completely disrupted at the cotyledon stage (Reverdatto et al., 2004). In addition to its role on the stability and translation of transcripts, ARE also has effects on transport and subcellular location (García-mauriño et al., 2017).

Found in 3’UTRs of short half-life transcripts, the sequence UGUUUGUUUGU (GU-rich element, GRE) is involved in the decay of mRNAs also mediated by CUGBP1 (Vlasova et al., 2008; Lee et al., 2010; Rattenbacher et al., 2010; Louis and Bohjanen, 2011). More importantly, Lee et al. (2010) demonstrated that GREs and AREs effects depend on the type of cell, suggesting a cell-specific context, and perhaps a cell-specific stage, since the effects of AREs were more significant in stem cells. This is probably due to different levels of CUGBP1 expression in each cell type, as well as APA events on target transcripts, suggesting that the same may occur for other *cis*-acting elements. Indeed, according to Kalsotra et al. (2008), the Elav-like family to which CUGBP1 belongs, is down-regulated during the development of the heart. More interestingly, the shortening of the 3’UTRs of CUGBP1 target mRNAs promoted by APA during the activation of human T cells resulted in a lower presence of GREs and increased levels of expression (Beisang et al., 2014).

Also found in 3’UTRs, a CU-rich sequence similar to ARE, seems to promote the instability of transcripts encoding the protein MARCKS (myristoylated alanine-rich C kinase substrate), possibly mediated by CUGBP1. However, the overexpression of two proteins of the Elav family, HuD and HuR, which bind with high affinity to CU-rich sequence promoted high levels of *MARCKS* expression (Wein et al., 2003). Similar results were found involving the ARE and HuD element in long 3’UTRs of the transcript from brain-derived neurotrophic factor (BDNF) in mouse neurons (Allen et al., 2013), and also for ARE and HuR in HEK293 cells, but an interaction between ARE and ZFP36 promoted degradation of transcripts (Mukherjee et al., 2014). This suggests that like ARE, the CU-rich *cis*-acting element is also the target of stabilizing and destabilizing RBPs. Despite this, 3’UTRs can have both stabilizing and destabilizing sequences. For example, the KRAS 3’UTR has sequences that bind stabilizing factors, such as HuR, but also has inhibitory sequences that are targets of microRNAs (Kim et al., 2016).

The downstream element (DST), highly conserved in a special gene family encoding small auxin up RNA (SAUR) from plants, consists of repetitions of the ATAGAT and GTA motifs located in the 3’UTR of mRNA (McClure et al., 1989; Newman et al., 1993). Studies involving mutation in these regions have shown that any change in one of these different motifs is enough to increase the stability of the mRNA (Sullivan and Green, 1996; Johnson et al., 2000). Mutations of the Pumilio response element (PRE) and ARE present in 3’UTRs of *her1* also dramatically increased the expression levels of the reporter gene. Mutations in ARE alone showed a slight increase in expression, compared to the double mutant PRE and ARE, suggesting that the two elements mediate the decay of mRNAs in parallel (Tietz et al., 2020). The G3A element, GA-rich, located in the 3’UTRs of the chicken elastin mRNA, and confirmed also for other animal species, showed stabilizing effects on the transcripts (Hew et al., 2000). Consistent with this finding, the deletion of a GA-rich sequence upstream of a NUE from the extensin 3’ regulatory region resulted in a reduction of up to 60% in the expression of the target gene in tobacco (Rosenthal et al., 2018).

As revealed by Geisberg et al. (2014), mRNAs with U-rich 3’UTRs also have high stability. Furthermore, it was found that the interaction of the poly-A tail with these U-rich sequences results in the formation of secondary structures (stem-loop and others double-stranded structures) with positive effects on the stability of transcripts. Recently, it was demonstrated that the AT-rich interactive domain-containing protein 5a (Arid5a) stabilizes the *OX40* (TNFR) transcripts in Th17 cells, through its interaction with a stem-loop formed by the GU-rich element (ADE-like) present in 3 ‘UTRs of *OX40* mRNA (Hanieh et al., 2018). A recent extensive analysis of Zebrafish 3’UTRs using UTR-seq has shown that U-rich (poly-U) and UUAG sequences are involved with stability and GC-rich sequences with instability (Rabani et al., 2017). The C-rich element (CRE) has also been shown to mediate transcript stability. The presence of CRE in 3’UTRs of the mu-opioid receptor (MOR) proved to be determinant for the stabilization of *MOR* transcripts. Depletion of Poly (rC) binding protein 1 (PCBP1), a CRE ligand, had negative effects on *MOR* mRNAs half-life. It has been suggested that when interacting with CRE, PCBP1 recruits other RPBs, such as AUF1 and PABP, with this complex being responsible for stabilizing *MOR* transcripts (Hwang C. K. et al., 2016).

Some RBPs are involved in translational repression. PUMs, are ligands of the PRE element, and are present in different organisms. After inserting PRE into the 3’UTR of the reporter gene for expression in HEK293 cells, PUMs have been shown to promote translational repression by a highly conserved deadenylation pathway, involving the recruitment of the CCR4-NOT (CNOT) complex. Interestingly, there was also a repression by PUMs independent of deadenylation (Etten et al., 2012). In addition, PUMs anchored to 3’UTRs can recruit argonaut and repress the translational activity of eEF1A (Friend et al., 2012). PUMs may also promote translational repression of reporter mRNAs in a PABP-dependent manner, probably preventing the interaction between PABP and EIF4G (Weidmann et al., 2014).

TIAR, an ARE-binding, has been shown to promote translational repression of eIF4A, eIF4E, eEF1B and c-Myc, being found in the 3’UTRs of all these factors (Mazan-mamczarz et al., 2006). A translational repression mediated by the cytoplasmic polyadenylation element (CPE) was also observed. Interestingly, the effects on translational repression are dependent on the proximity of CPE to PAS in the 3’UTRs (Dai et al., 2019). The presence of a CU-rich sequence in the 3’UTR of 15-lipoxygenase (*LOX*) gene has been identified as a mediator of translational silencing. The interaction of hnRNPK and hnRNPE1 with these sequences prevents the initiation of translation of *LOX* mRNAs by blocking the assembly of the 80S ribosome during the erythropoiesis process (Ostareck et al., 1997). Other forms of translational repression involving preventing the recruitment of subunits or blocking the assembly of ribosomal complexes, mediated by *cis*-acting elements and RBPs, have been reported in the literature (Duncan et al., 2006; Deng et al., 2008; Hussey et al., 2011). In fact, *cis*-acting elements are also found in 5’UTRs, which can act alone and even overlap effects conferred by *cis*-acting elements from 3’UTRs, and vice versa (Theil et al., 2018).

In addition, *cis*-acting elements and RBPs are also involved in transport, determining the location of mRNAs (Kislauskis et al., 1994). Equally, *cis*-acting elements are involved in determining tissue location during developmental stages (Bullock and Ish-horowicz, 2001). This allows for fine regulation of gene expression by promoting an asymmetric distribution of mRNAs, which is essential for cell polarization, division and motility, especially during embryonic development (Martin and Ephrussi, 2009; Zappulo et al., 2017). The construction of chimeric reporters demonstrated that 3’UTR sensorin promotes the localization of mRNAs to distal sensory neurites, although *cis*-acting elements have not been identified in the 3’UTR (Meer et al., 2012). The presence of localization elements (LEs) in 3’UTRs of approximately one-third of known dendritic mRNAs have been shown to be potent regulators of the location of *PSD-95* and *CaMKIIa* mRNAs (Subramanian et al., 2011).

Plants also have *cis*-acting elements involved in determining the location of mRNAs. Rice prolamine transcripts are preferentially located in the protein body of the endoplasmic reticulum (ER), mediated by a putative LE present in its 3’UTR. The construction of a chimeric construct showed that the 3’UTR of the prolamine is sufficient to direct the reporter mRNA to protein body ER (Hamada et al., 2003). Rice glutelin also has a putative LE in its 3’UTR that determine its location in the cisternal endoplasmic reticulum (ER), since the 3’UTR of glutelin was sufficient to promote ER cisternal localization of the reporter mRNA (Washida et al., 2009). More recently, it has been demonstrated that the determination of the location of prolamine and glutelin is dependent on two RBPs, RBP-P and RBP-L (Tian et al., 2018; Tian et al., 2019). Indeed, *cis*-acting elements can also predispose mRNAs to degradation by promoting a cytoplasmic or even tissue localization that is not suitable for the stability or translation of a particular transcript (Ding et al., 1993; Semotok et al., 2005; Tadros et al., 2007).

Additionally, it was observed that *cis*-acting elements and RBPs are involved in determining protein localization. Berkovits and Mayr (2015) proposed that during the translation process 3’UTRs can function as a scaffold, promoting the interaction of HuR with SET and the nascent amino acids. This interaction between SET and nascent amino acids determines the location of the newly synthesized protein in a RAC1-dependent manner. HuR depletion led to a reduction in surface CD47, CD44, ITGA1, and TNFRSF13C expression. Isoforms with long 3’UTRs from these four proteins showed U-rich sequences, which are probable targets for HuR.

## Plant 3’ Regulatory Regions for Expression of Target Genes

A careful selection of modulators of gene expression, such as 3’ regulatory regions, has proven to be an indispensable strategy when the goal is to maximize expression. Although 3’ regulatory regions are extremely important for gene expression, they are still poorly studied compared to other regulatory sequences (Ingelbrecht et al., 1989; Luo and Chen, 2007; Yang et al., 2009; Hirai et al., 2011; Hiwasa-Tanase et al., 2011; Rosenthal et al., 2018). Due to their great potential, 3’ regulatory regions such as *NOS* and *OCS* of *A. tumefaciens* and *35S* of cauliflower mosaic virus (CaMV) are widely used in plant expression vectors. Similar to *NOS* and *OCS*, the 3’ regulatory region of *35S* has *cis*-elements involved in cleavage and polyadenylation (Mogen et al., 1990; Macdonald et al., 1991; Sanfaçon et al., 1991). Other viral 3’ regulatory regions, such as the figwort mosaic virus (FMV) and the rice tungro bacilliform virus (RTBV), also have efficient polyadenylation signals, although they are less used in plant molecular biotechnology (Hay et al., 1991; Sanfaçon, 1994).

Indeed, *35S* has greater potential in regulating expression than *NOS* and *OCS*, both in monocot (rice) or dicot (tobacco) plants (Mitsuhara et al., 1996; Nagaya et al., 2010). However, although the number of 3’ regulatory regions identified and validated in plants is still reduced, several studies have shown that plant 3’ regulatory regions have a higher potential to increase expression compared to *NOS*, *OCS* or *35S* 3’ regulatory regions (**Table 1**) (Richter et al., 2000; Weeks et al., 2008; Yang et al., 2009; Nagaya et al., 2010; Hirai et al., 2011; Hiwasa-Tanase et al., 2011; Schaart et al., 2011; Kurokawa et al., 2013; Limkul et al., 2015; Diamos et al., 2016; Diamos and Mason, 2018; Yamamoto et al., 2018; Pérez-González and Caro, 2018; Rosenthal et al., 2018).

**Table 1:** Expression levels of reporter genes regulated by 3’ regulatory regions from plant genes compared to 3’ regulatory regions of *NOS*, *OCS* and *35S* widely used in plant molecular biotechnology.

**Plant 3’ regulatory regions**	**Expression levels of the reporter gene**	**Organism**	**Transformation Method**	**Expression System**	**References**
***Flaveria bidentisMe1***	64 to 440-fold higher than *OCS*	*F. bidentis*	*A. tumefaciens*	Stable	Marshall et al., 1997
**Potato *pinII***	~ 10-fold higher than *NOS*	Potato	*A. tumefaciens*	Stable	Richter et al., 2000
***F. bidentisMe1***	2 to 26-fold higher than *OCS*	Tobacco	*A. tumefaciens*	Stable	Ali and Taylor, 2001
***F. bidentisMe1***	~ 4-fold higher than *NOS*	Tobacco	*A. tumefaciens*	Stable	Ali and Taylor, 2001
***F. bidentisMe1***	~ 3-fold higher than *OCS*	Tobacco	*A. tumefaciens*	Stable	Schünmann et al., 2003
***R*ice *GluB-1***	4-fold higher than *NOS*	Rice	*A. tumefaciens*	Stable	Yang et al., 2009
***A. thaliana*** ***HSP***	~ 2,5-fold higher than *OCS*	Arabidopsis and Rice	*A. tumefaciens*	Transient	Nagaya et al., 2010
***A. thaliana*** ***HSP***	~ 2-fold higher than *NOS* and *35S*	Arabidopsis and Rice	*A. tumefaciens*	Transient	Nagaya et al., 2010
***A. thaliana*** ***ADH***	~ 1,4-fold higher than *NOS*	Arabidopsis	*A. tumefaciens*	Transient	Nagaya et al., 2010
***A. thaliana*** ***UBQ5***	~ 1,4-fold higher than *NOS*	Arabidopsis	*A. tumefaciens*	Transient	Nagaya et al., 2010
***A. thaliana*** ***HSP***	~ 8-fold higher than *NOS*	Tomato	*A. tumefaciens*	Stable	Hirai et al., 2011
**Apple *rbcS***	3 to 11-fold higher than *NOS*	Apple and tobacco	*A. tumefaciens*	Stable	Schaart et al., 2011
***Richadella dulcificaMIR***	1.5-fold higher than *NOS*	Tomato	*A. tumefaciens*	Stable	Hiwasa-Tanase et al., 2011
***Rice GluA-2***	2,45-fold higher than *NOS*	Rice	*A. tumefaciens*	Transient and Stable	Li et al., 2012
***Rice GluB-5***	3,12-fold higher than *NOS*	Rice	*A. tumefaciens*	Transient and Stable	Li et al., 2012
***Rice GluC***	2,14-fold higher than *NOS*	Rice	*A. tumefaciens*	Transient and Stable	Li et al., 2012
***A. thaliana*** ***HSP***	~7-fold higher than *NOS*	Tomato	*A. tumefaciens*	Stable	Kurokawa et al., 2013
***A. thaliana*** ***HSP***	~2-fold higher than *NOS*	*Nicotiana benthamiana*	*A. tumefaciens*	Stable	Limkul et al., 2015
**Potato *pinII***	8.5-fold higher than *NOS*	*N. benthamiana*	*A. tumefaciens*	Stable	Diamos and Mason, 2018
**Pea *rbcS***	5.4-fold higher than *NOS*	*N. benthamiana*	*A. tumefaciens*	Stable	Diamos and Mason, 2018
***A. thaliana*** ***HSP***	2.5-fold higher than *NOS*	*N. benthamiana*	*A. tumefaciens*	Stable	Diamos and Mason, 2018
***N. benthamianaNbHSP***	6.3-fold higher than *NOS*	*N. benthamiana*	*A. tumefaciens*	Stable	Diamos and Mason, 2018
***N. benthamianaNbACT3***	8.9-fold higher than *NOS*	*N. benthamiana*	*A. tumefaciens*	Stable	Diamos and Mason, 2018
**Tobacco *Ext* without native intron**	13.9-fold higher than *NOS* and 2.8-fold higher than *35S*	*N. benthamiana*	*A. tumefaciens*	Transient	Rosenthal et al., 2018

3’ regulatory regions: NOS (nopaline synthase), 35S (cauliflower mosaic virus), OCS (octopine synthase), HSP (heat shock protein), MIR (miraculin), rbcS (ribulose-1,5-biphosphate carboxylase), Glu (glutelin), Me1 (malic enzyme 1), Ext (extensin), pinII (proteinase inhibitor II), NbHSP (N. benthamiana heat shock protein), NbACT3 (N. benthamiana actin 3), ADH (alcohol dehydrogenase), UBQ5 (ubiquitin 5).

In alfalfa seedlings, constructions of the ribulose-1,5-bisphosphate carboxylase (*rbcS*) 3’ regulatory region was able to regulate higher levels of expression of the *GUS* reporter gene than the *NOS* using the *FMV 35S* promoter of the FMV (Weeks et al., 2008). Tobacco plants transformed with different combinations of promoters and 3’ regulatory regions, also demonstrated that the *rbcS* 3’ regulatory region results in higher levels of expression compared to *NOS* (Schaart et al., 2011). Similar results were also found for the pea *rbcS* 3’ regulatory region in *Nicotiana benthamiana* (Diamos and Mason, 2018). The regulation ensured by suitable 3’ regulatory regions has also been shown to be efficient for application in new genomic editing technologies, such as the Clustered Regularly Interspaced Short Palindromic Repeat Associated Cas9 Nuclease (CRISPR/Cas9) system. The use of the *Pisum sativum* *rbcS* *E9* 3’ regulatory region showed higher Cas9 levels in *A. thaliana* egg cells than *NOS* (Wang et al., 2015). In particular, it has already been demonstrated that the *rbcS E9* 3’ regulatory region has multiple putative PAS downstream of the canonical PAS (Hunt, 1988). Sequences identified mainly upstream of these PAS in the *rbcS E9* 3’ regulatory region, FUE and NUE elements, were determinant for the functionality and choice of these PAS. Interestingly, a single FUE appears to be involved in choosing three out of four different PAS (Hunt and Macdonald, 1989). Similar results were found for the 3’ regulatory region of the maize gene encoding (Wu et al., 1993) and of a wheat gene encoding a histone 3 (Ohtsubo and Iwabuchi, 1994).

In rice seeds, the accumulation of a modified house dust mite allergen (*mDer f 2*) was 4 times higher in constructions with the 3’ regulatory region from *glutelin* *B-1* (*GluB-1*), compared to the *NOS* (Yang et al., 2009). Similarly, *GluB-5*, *GluA-2* and *GluC* 3’ regulatory regions, also resulted in high levels of expression compared to *NOS* in rice (Li et al., 2012). As revealed by Yang et al. (2009), mRNAs extracted from seeds and leaves regulated by *NOS* showed a higher diversification in the choice of the PAS than the *GluB-1* 3’ regulatory region. This intense APA observed in *NOS* may be because the availability and/or diversity of subunits of the CPMC may vary according to the cell type, degree of cell differentiation, stage of development or in response to environmental changes (Hunt et al., 2008; Rao et al., 2009; Thomas et al., 2012; Lackford et al., 2014; Yu et al., 2019). Interestingly, Hiwasa-Tanase et al. (2011), studying recombinant *GUS* expression in tomatoes under the regulation of the *R. dulcifica MIR* 3’ regulatory region (miraculin), found that the PAS used were close to those that occur in native *MIR* mRNA. In this study, the levels of *GUS* expression using the *MIR* 3’ regulatory region were also higher compared to *NOS*.

In rice cells, the *GUS* or *Renilla Luciferase* (*Rluc*) expression driven respectively by the constitutive *CaMV 35S* promoter and the *elongation factor 1α* promoter, were 2-fold higher, when the *NOS* was replaced by the heat shock protein (*HSP*) 3’ regulatory region. This demonstrates that expression regulation by the *HSP* 3’ regulatory region is not affected by the promoter or reporter gene, although studies have shown that there seems to be an ideal combination between 3’ regulatory region and promoters. In addition, the *HSP* 3’ regulatory region showed higher levels of expression of *Rluc*, both in monocot and dicot, compared to the *NOS*, *OCS* or *35S* 3’ regulatory regions (Nagaya et al., 2010). Similar results were also found by Kurokawa et al. (2013) and Limkul et al. (2015).

In tomato fruits, the expression of recombinant *MIR* using the *35S* promoter was 6 to 8-fold higher when the *NOS* was replaced by the *HSP* 3’ regulatory region from *A. thaliana*. (Hirai et al., 2011). Interestingly, the concentration of recombinant *MIR* varied widely from tissue to tissue, both for *NOS* and for the *HSP* 3’ regulatory regions (Hirai et al., 2011). These differences probably also reflect the availability and tissue diversity of CPMC and other RBPs factors involved in post-transcriptional processes. Perhaps it is also due to APA events, allowing fine regulation of expression (Takagaki et al., 1996; Tang et al., 2002; Quesada et al., 2003; Simpson et al., 2003; Delaney et al., 2006; Dong et al., 2007; Ji et al., 2009; Tian and Manley, 2013). Matsui et al. (2014) demonstrated that the use of a longer version of the *HSP* 3’ regulatory region results in higher levels of expression compared to a smaller version. According to the authors, the high levels of expression achieved in the longer version occurred due to the presence of a *matrix attachment region* (*MAR*), AT-rich DNA sequences that assist in the chromatin structural organization, being involved in transcriptional control (Abranches et al., 2005; Tetko et al., 2006).

As revealed by Pérez-González and Caro (2018), increases in expression of firefly luciferase (*LUC*) regulated by the *HSP* 3’ regulatory region compared to the *35S*, in part, occurred due to less promoter methylation, through a phenomenon called RNA-directed DNA methylation (RdDM). It has been proposed that siRNA produced by PTGS events triggers RdDM, probably involving non-polyadenylated transcripts, resulting from readthrough or improperly terminated mRNA. In *N. benthamiana*, Diamos and Mason (2018) found that the expression directed by some plant 3’ regulatory regions were 2.5- to 8.9-fold higher than the *NOS* (**Table 1**). Similarly, the use of other *A. thaliana* 3’ regulatory regions, a*lcohol dehydrogenase* (*ADH*), *histone H4* (H4), and *ubiquitin 5* (*UBI5*), also resulted in higher levels of *GUS* activity compared to *NOS* (Nagaya et al., 2010). Potato plants transformed to express recombinant *hepatitis B surface antigen* (*HBsAg*) showed higher levels of *HBsAg* mRNA when soybean *VSP* gene or potato *pinII* genes 3’ regulatory regions were used, compared to the *NOS* (Richter et al., 2000).

Also, the *Me1* gene 3’ regulatory region from *F. bidentis* was able to increase *GUS* expression several times compared to the *35S* or *OCS* 3’ regulatory regions in leaves of *F. bidentis* plants (Ali and Taylor, 2001). The use of the tobacco *extensin* 3’ regulatory region (*Ext*) to express different recombinant reporter proteins in *N. benthamiana* leaves resulted in high levels of expression compared to the *NOS* and *35S*, even higher than the *VSP* 3’ regulatory region. The *Ext* 3’ regulatory region has been shown to prevent readthrough, resulting in high concentrations of recombinant mRNAs and proteins (Diamos et al., 2016). It is worth to note that the presence of a native intron in the *Ext* tobacco 3’ regulatory region appears to have deleterious effects on expression (Rosenthal et al., 2018).

Rosenthal et al. (2018), demonstrated that the removal of this intron results in levels of expression up to 3-times higher compared to *Ext* in its native form, and much higher than *NOS* (13.5 x), *VSP* (11.9 x) and *35S*, although to a lesser degree (2.8 x). Constructions with *Ext* without intron showed low or undetectable readthrough, and among the 5 NUEs, about 75% of the polyadenylation events observed occurred from the fourth element. These results are in agreement with the expression levels of a shorter version of *Ext* without intron that lost this main NUE. In fact, a version of *Ext* without intron, lacking its first 465 nt deleted, showed no expression, probably due to the loss of a putative FUE, demonstrating the importance of this *cis*-element for gene expression. On the other hand, the presence of a native intron in the maize *Hrgp* 3’ regulatory region had positive effects on the regulation of expression in maize compared to the 3’ regulatory region in its native conformation (Menossi et al., 2003).

Furthermore, the use of double 3’ regulatory regions has resulted in considerable increases in expression levels compared to the use of a single 3’ regulatory region (Nagaya et al., 2010). According to Luo and Chen (2007), the cloning of two 3’ regulatory regions, *35S* and *NOS* downstream from *GUS*, was enough to reduce the levels of readthrough transcripts and siRNA, as well as increased *GUS* expression. Similar results were found by Beyene et al. (2011). In the same way, the use of two 3’ regulatory regions, *Ext* without a native intron and *NbACT3*, resulted in 2.8-4 times higher expression levels compared to the use of a single 3’ regulatory region (Diamos and Mason, 2018). Likewise, the combination of two downstream 3’ regulatory regions of *GFP*, *Ext*, and *HSP* in *N. benthamiana* leaves, resulted in expression levels of 1.7-2.2-fold higher compared to the use of a single 3’ regulatory region. On the other hand, the use of three 3’ regulatory regions appears to result in low levels of expression (Yamamoto et al., 2018).

Finally, some examples of the application of the 3’ regulatory region in plant biotechnology that have revolutionized agriculture are listed. The first transgenic *Bt* (*Bacillus thuringiensis*) plant produced, tomato, in 1987, used the *NOS* 3’ regulatory region (Fischhoff et al., 1987). The *35S* has been used in the generation of *Bt* maize (Koziel et al., 1993). The rice event GR2E (Golden Rice) plants express three transgenes to increase beta-carotene levels, and all the genes use the *NOS* regulatory region (Paine et al., 2005). The Bollgard Cotton plants have a gene encoding a *Bt* protein, using a plant 3´regulatory region from the α’ subunit of β-conglycinin gene (Perlak et al., 1990; Biosafety Clearing House, 2006). Likewise, the *NOS* 3’ regulatory region was used in the production of the first glyphosate-resistant plants, such as soybeans and wheat (Guilan et al., 2002; Parrot and Clemente, 2004). More recently, the *rbcS E9* 3’ regulatory region has also been used in the generation of glyphosate resistant soybeans (Malven et al., 2015). In addition, transgenic plants have been developed to obtain biomolecules for therapeutic purposes (Chen and Davis, 2016), including, but not limited to antigens, antibodies, epitopes of antigens, coagulation factors and antimicrobial, employing different 3’ regulatory regions, such as *NOS*, *VSP*, *pinII*, and *psbA* from lettuce (Richter et al., 2000; Giritch et al., 2006; Rajabi-Memari et al., 2006; Schulz et al., 2015; Su et al., 2015a; Su et al., 2015b).

## Conclusions and Perspectives

Molecular biotechnology has enabled new ways to exploit the potential of plants. Firstly, the genetic improvement of several species has allowed significant increases in food production (Duvick, 1996; Gepts and Hancock, 2006). With rapid population growth, it is essential to create new strategies to increase food production, their nutritional content, and to reduce environmental impacts. The development of plants more resistant to abiotic and biotic stresses have allowed considerable increases in production, as well as a reduction in the use of pesticides and fertilizers, and even water. Secondly, the possibility of transforming plants into bioreactors has allowed the production of proteins for industrial applications, research, diagnosis, or therapeutic purposes, among others (Sharma and Sharma, 2009; Desai et al., 2010). Besides the production cost being much lower, there are several other advantages and even limitations to producing proteins in non-plant biological systems, such as microorganisms or animal cells (Streatfield, 2007; Desai et al., 2010; Shinmyo and Kato, 2010; Egelkrout et al., 2012; Chen and Davis, 2016).

The use of plant 3’ regulatory regions in the construction of vectors has shown to be able to not only optimize, but also to make possible a fine regulation of gene expression, presenting potential superior to 3’ regulatory regions, *NOS*, *OCS* and *35S*, commonly used in the production of genetically modified organisms (Mitsuhara et al., 1996; Yang et al., 2009; Nagaya et al., 2010; Diamos and Mason, 2018; Rosenthal et al., 2018), therefore, very useful in plant molecular biotechnology. However, the molecular mechanism behind optimization in expression remains poorly understood. We know that 3’ regulatory regions can have multiple *cis*-elements directly involved in the cleavage and polyadenylation steps, and, as observed in mammals, polyadenylation in plants certainly requires a very well-orchestrated interaction between *cis*-elements and CPMC.

Although the canonical NUE AAUAAA is present in more than half of mammalian genes, it is found in only 10% of plant genes, certainly due to the high NUE degeneration that plants tolerate (Rothnie et al., 1994; Loke et al., 2005), consistent with the findings of Hiwasa-Tanase et al. (2011). Analyzing the effects of four different lengths of the *MIR* 3’ regulatory region (46, 287, 508 and 1085 nt), these authors found almost the same expression levels for the transcripts containing 508 and 1085 nt. Interestingly, only the longest version had canonical NUEs, confirming that plant CPMC can recognize sequences similar to AAUAAA with a high degree of variation. Plant 3’ regulatory regions commonly have several NUE and PAS in their sequences (Nagaya et al., 2010; Hiwasa-Tanase et al., 2011; Rosenthal et al., 2018). The interaction of CPMC with these different NUE and PAS can lead to APA events, and, consequently produce mRNAs with different 3’UTR from the same 3’ regulatory region, resulting in different levels of expression, since 3’UTR can present *cis*-acting elements involved in the stability, transport and translation of mRNAs. In addition, weak PAS 3’ regulatory regions can promote APA events within exons, resulting in non-stop codon mRNAs, which are direct targets for silencing *via* nonstop decay (NSD) (Frischmeyer et al., 2002; Szádeczky-Kardoss et al., 2018).

In fact, transformed plants with constructions without a 3’ regulatory region have low transcriptional and translational transgene levels, or even no expression (Ingelbrecht et al., 1989; Luo and Chen, 2007). The absence of the 3’ regulatory region results in the choice of random PAS contained within the plants own genomic DNA, resulting in longer 3’UTR, which generally have *cis*-acting elements involved in mRNA decay (Ingelbrecht et al., 1989; Shi and Manley, 2015). Furthermore, the absence of 3’ regulatory region may lead to readthrough mRNA or abortive elongation, resulting in incorrectly terminated and non-polyadenylated transcripts, direct targets of RDR6-mediated PTGS (Luo and Chen, 2007; Baeg et al., 2017). In addition, mutated PAS can generate readthrough events, with RNA Polymerase II (RNAPII) sequestering factors involved in initiation and stretching, with negative effects on expression, consistent with the findings by Wei et al. (2017). Pérez-González and Caro (2018), also demonstrated that the absence of a 3’ regulatory region results in high methylation of the promoter through RdDM events, with negative effects on the expression of the target gene. It is likely that the use of 3’ regulatory regions with weak polyadenylation signals may also promote RdDM. It would be interesting to have a better understanding of the mechanisms that regulate these processes, starting with a more detailed exploration of the effects of pathways involved in gene silencing such as PTGS-RDR6 and PTGS-RdDM.

The effects of two 3’ regulatory regions for the expression of the target gene has also generated curious results, worthy of more attention. The use of double 3’ regulatory regions for expression of target genes not only increases expression, but also reduces readthrough events and prevents depletion *via* PTGS-RDR6, prolonging the half-life of mRNAs (Luo and Chen, 2007; Beyene et al., 2011). Intriguingly, reversing the position of the 3’ regulatory regions, *35S-NOS* to *NOS-35S*, there was a 40% loss in expression levels (Diamos and Mason, 2018). Possibly, this reduction occurred due to the positioning of strong PAS further downstream, when the inversion was made, resulting in longer 3’UTRs that maybe has *cis*-acting elements involved in the degradation of transcripts (Gutiérrez et al., 1999; Hui et al., 2003; Wein et al., 2003; Garneau et al., 2007; Vlasova et al., 2008; Lee et al., 2010; Allen et al., 2013; Mukherjee et al., 2014; Rabani et al., 2017; Tietz et al., 2020).

Additionally, it would be interesting to explore the effects of the presence of other regulatory sequences on expression, as in the case of *MARs* (matrix attachment regions) elements. The presence of *MARs* can interfere with the chromosomal DNA conformation, making genes more accessible for transcription, thus increasing expression rates. As revealed by Mlynárová et al. (2003), *MARs* can prevent the silencing of transgenes. Corroborating this finding, Matsui et al. (2014) reported that increases in expression levels shown by the long version of the *HSP* 3’ regulatory region were possibly due to the presence of a *MAR*, absent in the smaller version of the *HSP*. Similar results were found by Diamos and Mason (2018), using tobacco *MARs* RB7 and TM6, downstream of different 3’ regulatory regions, resulting in a 60-fold increase in expression compared to using only the 3’ regulatory region.

Another interesting point for further studies is related to the fact that in some cases the presence of introns in 3’ regulatory regions may have either positive or negative effects on expression (Chung et al., 2006; Bicknell et al., 2012). Probably, intronic regions can, as well as 3’UTRs, have *cis*-acting elements involved in mRNA metabolism, which may explain this controversy. The repetitive presence of dinucleotide CA in intronic sequences of nitric oxide endothelial synthase (eNOS) was linked to transcript stability, involving heterogeneous nuclear ribonucleoprotein L (hnRNP L). Interestingly, hnRNP L depletion results in reduced eNOS expression, as well as leads to APA in eNOS, suggesting that this CA-rich sequence it is also the target of pro-decay RBPs (Hui et al., 2003). Moreover, the presence of introns in 3’UTRs can promote the degradation of mRNA *via* nonsense-mediated decay (NMD) (Kertész et al., 2006; Schweingruber et al., 2013). Also, another point that may explain this controversy would be tissue specificity that some introns present (Sieburth and Meyerowitz, 1997; Menossi et al., 2003; Kooiker et al., 2005; Showalter et al., 2010).

Last, but not least, it would interesting be to investigate the effects of the ideal combination between 3’ regulatory regions and promoters. The expression levels of target genes can vary according to the combination of 3’ regulatory regions and promoters. The combination of strong 3’ regulatory regions with strong promoters reduces expression, whereas a combination of weak 3’ regulatory regions with strong promoters, or the opposite, increased expression (Li et al., 2012; Wei et al., 2017). Also, it would be interesting to identify whether and which 3’ regulatory regions present tissue specificity (Debode et al., 2013), widely observed in promoters, as noted by Yang et al. (2009); Hirai et al. (2011); Kurokawa et al. (2013). Indeed, APA events are involved in the regulation of tissue-specific development (Chakrabarti et al., 2018), which may present a higher frequency than splicing events (Wang et al., 2008). This regulation is likely to involve tissue diversity of CPMC factors, as well as inhibitors. However, to test this hypothesis, it will be essential to identify all subunits of the plant CPMC, as well as to know its interaction network, how this network interacts with *cis*-elements and with possible inhibitors or stimulators. It would also be important to know whether, in fact, the levels and diversity of CPMC subunits change from tissue to tissue, with the stage of the cell cycle or development and also with the cellular state. These and other important questions remain to be answered in future studies.

## Author Contributions 

Drafting the work and manuscript writing: WB. Critical review and manuscript writing: MM.

## Funding 

This work was funded by São Paulo Research Foundation (FAPESP), research grant 2013/15576-5 and 2014/50884-5 (MM). WB received a fellowship from Brazilian Federal Agency for Support and Evaluation of Graduate Education (CAPES, Brazil; 88882.329484/2019-01) and MM was recipient of a Research Fellowship from National Council for Scientific and Technological Development (CNPq, Brazil; Grant #333270/2018-7).

## Conflict of Interest

The authors declare that the research was conducted in the absence of any commercial or financial relationships that could be construed as a potential conflict of interest.
